# Network analysis of sleep quality-anxiety-depressive symptoms among patients with chronic heart failure

**DOI:** 10.3389/fpsyt.2026.1734383

**Published:** 2026-02-25

**Authors:** Tingting Liao, Yanmei Gan, Yao Du, Wenhua Huang, Gaoye Li

**Affiliations:** 1 Department of Cardiovascular Medicine, The First Affiliated Hospital of Guangxi Medical University, Nanning, China; 2 Department of Radiation Oncology, The First Affiliated Hospital of Guangxi Medical University, Nanning, China

**Keywords:** anxiety and depressive symptoms, chronic heart failure, network analysis, sleep quality, symptom network

## Abstract

**Objective:**

While recent studies in related populations have confirmed the co-occurrence of sleep and mental symptoms in cardiac conditions, the specific network structure connecting these symptoms in a dedicated CHF cohort has not been fully elucidated. This study employed network analysis to identify central and bridging symptoms within this network.

**Methods:**

A cross-sectional study was conducted among 406 patients with chronic heart failure (CHF) at a hospital in Nanning, Guangxi, China, between August 2024 and March 2025. All participants completed a questionnaire assessing general and disease-specific characteristics. The Pittsburgh Sleep Quality Index (PSQI) was used to assess sleep quality, while anxiety and depressive symptoms were evaluated using the Hospital Anxiety and Depression Scale (HADS). A network analysis was conducted using Gaussian Graphical Models (GGM) in R software to explore the interrelationships among symptoms. Centrality indices and bridge expected influence (BEI) were used to identify central and bridge symptoms, respectively.

**Results:**

Analysis of the network centrality indicated that the HADS12 (not looking forward with enjoyment to things) and HADS13 (sudden panic) categories showed the highest centrality, with values of 1.205 and 1.208, respectively, showing their strong linkage with other symptoms and functions as central nodes in the network. Furthermore, PSQI-A (subjective sleep quality) was identified as the key bridge symptom (bridge expected influence=0.541), indicating its primary role in connecting the sleep and psychological symptom clusters.

**Conclusions:**

The identification of core and bridging symptoms in the CHF-specific symptom network underscores the potential for targeted interventions. Addressing these critical symptoms may prove beneficial in improving treatment outcomes and enhancing the quality of life for CHF patients, particularly those with comorbid anxiety and depressive symptoms.

## Introduction

Recent epidemiological studies have shown that chronic heart failure (CHF), a complex clinical syndrome, affects >56 million individuals globally. This figure is estimated to rise by 46% by 2030 (1), presenting significant challenges not only for healthcare systems but also for affected individuals, especially in terms of quality of life and mental well-being. This projected increase in CHF prevalence represents a critical challenge in contemporary public health governance. Sleep disorders are defined as persistent abnormalities in sleep patterns or physiological dysfunction related to sleep, usually manifested as poor sleep quality. These impact adversely on normal physiological requirements in terms of sleep quality, sleep duration, and maintenance of the circadian rhythm, leading to functional impairment and subjective distress during daytime (2). It has been reported that approximately 85.8% of patients with CHF experience poor sleep quality (3). Studies (4, 5) have also shown that poor sleep quality in patients with CHF was independently associated with adverse clinical outcomes, including significantly impaired quality of life, the development of anxiety and depressive symptoms, a progressive decline in physical functioning, persistent fatigue, and elevated 30-day readmission rates.

Previous studies have demonstrated a relationship between poor sleep quality and the presence of anxiety and depressive symptoms in individuals diagnosed with CHF (6, 7). The prevalence of anxiety and depressive symptoms is significantly higher in the CHF population. It was found that approximately 21.5% of patients with CHF had symptoms of clinical depression, while close on 30% of patients reported anxiety levels reaching clinically significant levels (8). These mental health issues may be related to lifestyle changes associated with CHF, as well as the uncertainty surrounding the progression of heart failure (9). A study by Palagini et al (10) identified a significant correlation between increased severity of anxiety and depressive and the primary clinical outcomes of CHF, including hospitalization due to heart failure, cardiovascular-related mortality, and the need for interventions such as heart transplantation or the implantation of left ventricular assist devices. There is a reciprocal relationship between sleep quality and anxiety-depressive symptoms. Numerous studies have demonstrated that poor sleep quality is a key predictor of the development of anxiety and depressive symptoms. It has been found that individuals experiencing chronic sleep disturbances are predisposed to the development of mental health issues (11, 12). Conversely, the presence of anxiety and depressive symptoms can also lead to or worsen compromised sleep quality, creating a vicious cycle (13). When people are anxious or depressed, their minds may be filled with excessive worry, negative thoughts, or emotional turmoil, which can directly disrupt the normal sleep cycle (14). For instance, these individuals may struggle to fall asleep, experience recurrent awakenings throughout the night, or awaken prematurely in the morning. However, it is noteworthy that effective interventions for poor sleep quality can, to a large extent, prevent the onset of anxiety and depressive symptoms, providing strong support for the maintenance of mental health (15). These strategies include behavioral therapies, such as insomnia-focused cognitive behavioral therapy (CBT-I), which assist individuals in the recognition and modification of detrimental thought patterns and sleep-related behaviors. Lifestyle modifications, such as maintaining a consistent sleep routine, optimizing the sleep environment for comfort, and limiting consumption of caffeine and alcohol, are also recommended (16, 17).

Network analysis is a novel data analysis method that overcomes the limitations of traditional data analysis by its ability to represent features and information in the form of a comprehensive network system (18). The system is composed of a network structure comprising “nodes” and “edges”, which are then used to identify interactions and relationships among factors. Network analysis has three core advantages, namely, its ability to reveal microscopic correlations among variables, visualize dynamic interaction patterns, and quantify the influence of nodes based on centrality indicators (19). Network analysis can determine interactions among symptoms in the symptom clusters of mental disorders, and can also quantify the relationships among individual symptoms (20). It is also able to identify interactions among different symptoms and groups of symptoms and determine core symptoms in the network structure. This approach not only helps to explore the mechanisms underlying internal and mutual influences among mental disorders but can also identify suitable interventions for preventing to treating diseases, thus having significant practical value (21, 22).

Although the co-occurrence of these symptoms is recognized, previous studies (7, 23–25) relying on traditional statistical methods (e.g., correlation or regression) is limited in its ability to model the complex interactions among specific symptoms, as evidenced by studies (25) such as Le Grande et al. (2016) which established their longitudinal links. These methods typically treat symptoms as independent outcomes or aggregate them into total scores, thereby obscuring the fine-grained relational structure (26–30). Network analysis addresses this gap by conceptualizing symptoms as a system of direct interactions, allowing us to identify the most central and interconnected symptoms. Consequently, without this network approach, treatment strategies may lack precision by failing to prioritize these key drivers of comorbidity.

Network analysis, by modeling these interactions as a system of interconnected nodes, offers a more comprehensive and nuanced understanding of how symptoms influence one another, and can thus lead to more targeted interventions (31). Therefore, this study applied network analysis to examine the interplay between sleep quality, anxiety, and depressive symptoms in individuals suffering from CHF. By identifying core and bridge symptoms within the symptom network, we aim to elucidate the complex relationships between sleep quality and anxiety-depressive comorbidity in this population. The findings may inform integrated the use of treatment strategies to ultimately improve the sleep quality, mental health, and overall quality of life of patients with CHF.

## Materials and methods

### Study participants and design

This cross-sectional study was conducted from August 2024 to March 2025 at a tertiary Class-A hospital in Nanning, Guangxi, China, a major regional cardiovascular referral center. The inclusion criteria encompassed the following: (1) individuals aged 18 years or older; (2) a confirmed diagnosis of heart failure (HF) in accordance with the 2021 European Society of Cardiology (ESC) Guidelines for the Diagnosis and Management of Acute and Chronic Heart Failure; (3) being in a clinically stable condition while covering the spectrum of CHF severity from NYHA class II to IV; (4) the ability to complete questionnaires either independently or with verbal assistance; and (5) provision of written informed consent and voluntary participation in the study. Conversely, the exclusion criteria included (1): a history of severe comorbidities, such as malignant neoplasms, liver, kidney, or pulmonary failure (2); current diagnoses of severe mental health disorders (e.g., schizophrenia or bipolar disorder) or acute suicidal ideation (3); identified sleep disorders necessitating medical intervention (e.g., obstructive sleep apnea syndrome or chronic insomnia) (4); the use of psychoactive drugs (e.g., antidepressants, anxiolytics, or sedative-hypnotics) within the preceding two weeks; and (5) communication impairments that hindered completion of the questionnaires.

All researchers received training before data collection, and adopted a standardized protocol to verbally explain the study objectives and procedures to participants in person. Guided by the recommended sample sizes for network analyses (32), the required sample size was determined by exceeding the total number of model parameters, including threshold and pairwise relational parameters. Specifically, the threshold parameter corresponded to the number of nodes (21), and pairwise parameters were calculated as (nodes × (nodes − 1))/2, yielding 210 for 21 nodes. The enrollment of 406 participants satisfied these criteria.

### Measurement methods

#### Questionnaires used for determination of socio-demographic and clinical characteristics

Information on socio-demographic characteristics, such as sex, age, marital status, educational level, occupation, and monthly household income per capita, as well as clinical characteristics, including drinking history, smoking history, NYHA classification, coronary heart disease, myocardial infarction, valvular heart disease, hypertension history, diabetes mellitus, and prescribed medications, was obtained from the questionnaires. The socio-demographic characteristics were self-reported by patients, while clinical information was obtained from their medical records.

#### Pittsburgh sleep quality index

This study utilized the Chinese version of Pittsburgh Sleep Quality Index (PSQI) scale to assess sleep quality in patients with CHF over the past month. This scale was developed by Buysse et al (33) in 1989, and was translated by the Chinese researchers Liu et al. (34) in 1996. This version comprises 18 items across 7 components, each rated 0-3, yielding a global score ranging from 0 to 21. In accordance with the validation study of the Chinese version, a total PSQI score of ≥8 was used to define poor sleep quality. The Chinese version of the scale has demonstrated good internal consistency, with a Cronbach’s α of 0.88 in this population.

#### Hospital anxiety and depression scale

Anxiety and depressive symptoms were assessed using the Chinese version of Hospital Anxiety and Depression Scale (HADS). The HADS is a widely used 14-item instrument comprising two subscales: the anxiety subscale (HADS-A), composed of the odd-numbered items (items 1, 3, 5, 7, 9, 11, and 13), and the depression subscale (HADS-D), composed of the even-numbered items (items 2, 4, 6, 8, 10, 12, and 14), each containing 7 items. Each item is scored from 0 to 3, resulting in a subscale score ranging from 0 to 21. In accordance with the original scoring instructions, several positively worded items were reverse-scored so that higher scores consistently indicate greater symptom severity. Specifically, item HADS7 (feeling relaxed) in the anxiety subscale and items HADS2 (enjoyment), HADS4 (laughing and seeing the funny side of things), HADS6 (cheerfulness), HADS12 (looking forward with enjoyment to things), and HADS14 (enjoyment of reading or television) in the depression subscale were reverse-coded prior to analysis. Therefore, in **Table 1**, the content of these items is presented in terms of their reversed meanings to ensure conceptual consistency with the scoring direction (e.g., HADS2 “enjoyment” is expressed as “not enjoying the things that used to be enjoyed”). In line with the original validation and common practice, a subscale score of ≥8 was used to indicate the probable presence of clinically significant symptoms (35). The Chinese version of the HADS was evaluated in a cohort of 615 individuals diagnosed with cardiovascular disease, demonstrating good internal consistency, with an overall Cronbach’s α of 0.890, and subscale coefficients of 0.820 for anxiety and 0.807 for depression (36).

**Table 1:** Characteristics of the study participants (n = 406).

Variables		N(%)
Sex	male	264(65.0%)
	female	142(35.0%)
Age, years	18~59	131(32.0%)
	60~79	234(58.0%)
	≥80	41(10.0%)
Educational level	junior high school or below	300(73.9%)
	senior high school or above	106(26.1%)
Occupation	Farmers/Workers	204(50.2%)
	Employees of enterprises/institutions	70(17.2%)
	Retirees or others	132(32.5%)
Marital status	Married	257(63.3%)
	Others	149(36.7%)
Monthly household income per capita	≤5000RMB	280(69.0%)
	>5000RMB	126(31.0%)
Drinking history	None	182(44.8%)
	Yes	224(55.2%)
Smoking history	None	212(52.2%)
	Yes	194(47.8%)
NYHA classification	II	66(16.3%)
	III	165(40.6%)
	IV	175(43.1%)
Coronary heart disease	None	163(40.1%)
	Yes	243(59.9%)
Myocardial infarction	None	383(94.3%)
	Yes	23(5.7%)
Valvular heart disease	None	327(80.5%)
	Yes	79(19.5%)
Hypertension history	None	204(50.2%)
	Yes	202(49.8%)
Diabetes mellitus	None	305(75.1%)
	Yes	101(24.9%)
Number of prescribed medications.	≤2	36(9.0%)
	3~5	216(53.0%)
	≥5	154(38.0%)

#### Data analysis

Data were analyzed using SPSS version 27.0. Categorical variables are presented as cases and percentages, while normally distributed continuous variables are reported as mean ± standard deviation. If data is non-normally distributed, median (Inter-Quartile Range, IQR) will be presented. Furthermore, the network analysis was conducted using R software (version 4.2.3) to examine the interrelationships among the included symptoms. This study applied the Gaussian Graphical Model (GGM) to construct a sleep quality-anxiety/depressive symptom network for patients with CHF (37). The GGM was chosen because it is appropriate for the continuous data and, as a partial correlation network, the GGM depicts conditional dependence relationships between symptoms after controlling for all others, thereby helping to identify unique direct connections. Furthermore, the GGM is well-suited for the exploratory, cross-sectional nature of the study, as it robustly maps these interactions without relying on the strong and often unverifiable causal-direction assumptions required by other models, such as Bayesian networks (32). In addition, other modeling approaches such as Mixed Graphical Models (MGMs) and latent variable models was considered. MGMs are designed for mixed data types, which did not align with our exclusively continuous variables (38). Latent variable models operate on a common-cause paradigm (39), contrasting with our study’s network theory approach that emphasizes direct symptom interactions. For modeling conditional dependencies among continuous, cross-sectional symptom data without imposing latent constructs or causal assumptions, the GGM was the most parsimonious and theoretically coherent choice. The Fruchterman-Reingold layout was used for network representation. In this layout, edge thickness and color indicate partial correlations between the symptoms (40). A Spearman’s rank correlation matrix was computed to capture the associations between symptoms, as it is robust to non-normality. This matrix was then used as the input for network estimation. In the optimal sparse estimation stage of the partial correlation matrix, the Extended Bayesian Information Criterion (EBIC) and Graphical Least Absolute Shrinkage and Selection Operator (gLASSO) in the bootnet R package, namely the EBICgLASSO method (41) was applied. The γ hyperparameter in EBIC was set to 0.5, the default value that balances sensitivity and specificity while encouraging sparse, interpretable network structures. This method can accurately perform optimal sparse estimation of the partial correlation matrix to show the correlation structure between symptoms more clearly.

The primary symptoms of the network were assessed primarily through the central index, encompassing strength, closeness, betweenness, and bridge expected influence. Each of these indices can be used to evaluate node importance within the network. Strength centrality represents a cumulative measure of the absolute intensities linking a target symptom to every other symptom in the network. A higher value indicates that the symptom has more extensive and stronger connections with other symptoms. Close centrality quantifies the mean path length between a given symptom and all remaining symptoms, reflecting the efficiency of information transmission by that symptom in the network. Betweenness centrality quantifies how often a node lies on the shortest paths between other node pairs, highlighting its role as a mediator. Additionally, bridge expected influence (BEI) was used to identify bridge symptoms, which are nodes with strong connections across different symptom clusters (e.g., poor sleep quality and anxiety/depressive symptoms). A higher BEI value indicates a greater capacity to influence other clusters (18).

## Results

### Demographic and clinical characteristics

A total of 406 patients were enrolled in the study. Their ages were between 23 and 91 years, averaging 63.97 ± 13.48 years. Among them, 264 (65%) were male and 142 (35%) were female. The general information on the patients is shown in **Table 1**.

### Scores and occurrence of symptoms

The mean PSQI score of the study participants was 9.01 ± 4.25 points. Of the 406 participants, 233 (57.39%) had poor sleep quality. The mean score of the Anxiety Subscale in the HADS was 9.30 ± 5.83, with 245 patients (60.34%) reporting anxiety symptoms. The mean score of the HADS Depressive Subscale (HADS-D) was 10.78 ± 5.42, with 297 patients (73.15%) experiencing depressive symptoms. The scores of each item on the scale are detailed in **Table 2**.

**Table 2:** Score of each item in PSQI and HADS scales (n = 406).

Label	The content to be expressed	Mean (SD) or median (QR)
HADS		
HADS1	Tense or wound wp	1.33 ± 0.877
HADS2	Not enjoying the things that used to be enjoyed	1.55 ± 0.946
HADS3	Afraid something will happen	1.40 ± 0.975
HADS4	See the bad side of things	1.53 ± 0.937
HADS5	Worrying thoughts go through mind	1.28 ± 0.972
HADS6	Not feeling cheerful	1.57 ± 0.945
HADS7	Trouble relaxing	1.69 ± 0.977
HADS8	Feeling being slowed down	1.51 ± 0.885
HADS9	Having a frightened feeling like ‘butterflies’ in the stomach	1.00(1.00, 2.00)
HADS10	Lost interest in appearance	1.00(1.00, 2.00)
HADS11	Restlessness	1.00(1.00, 2.00)
HADS12	Not looking forward with enjoyment to things	1.00(1.00, 2.00)
HADS13	Sudden panic	1.00(0.00, 2.00)
HADS14	Not enjoying books or program	2.11 ± 1.045
PSQI		
A	Subjective sleep quality	1.95 ± 0.948
B	Sleep latency	1.81 ± 0.865
C	Sleep duration	1.00(1.00, 1.00)
D	Habitual sleep efficiency	1.00(0.00, 2.00)
E	Sleep disturbances	1.36 ± 0.695
F	Use of sleeping medicine	0.00(0.00, 0.00)
G	Daytime dysfunction	1.46 ± 0.992

### Network of sleep quality-anxiety/depressive symptoms in patients with CHF

**Figure 1:**
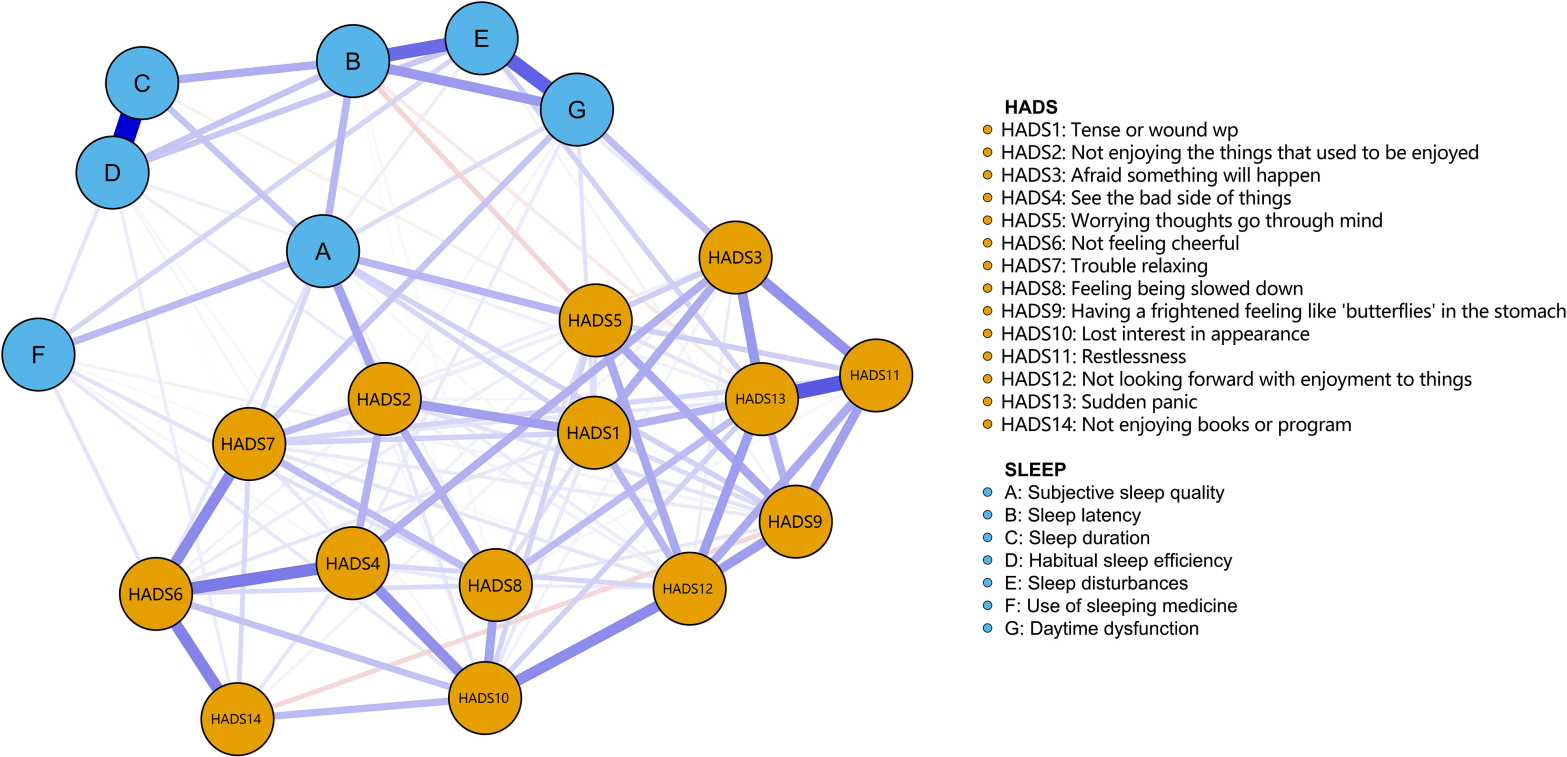
Symptom-based network of comorbid sleep quality-anxiety/depressive symptom in patients with CHF. (Nodes indicate manifestations of sleep qualityanxiety/depressive symptom. Edges represent associations. Solid blue edges indicate positive correlations, while dashed red edges represent negative correlations. Edge thickness corresponds to the associated intensity.).

In the sleep disorders-anxiety/depressive symptom network, among 210 potential edges, 118 (56.2%) non-zero edges were observed. The mean edge weight was 0.04. The strongest correlation were observed with HADS11 (restlessness) and HADS13 (sudden panic), followed by HADS4 (see the bad side of things) and HADS6 (not feeling cheerful). In terms of sleep disorders, the strongest correlation were found with C (sleep duration) and D (habitual sleep efficiency), followed by E (sleep disturbances) and G (daytime dysfunction) (**Figure 1**).

**Figure 2:**
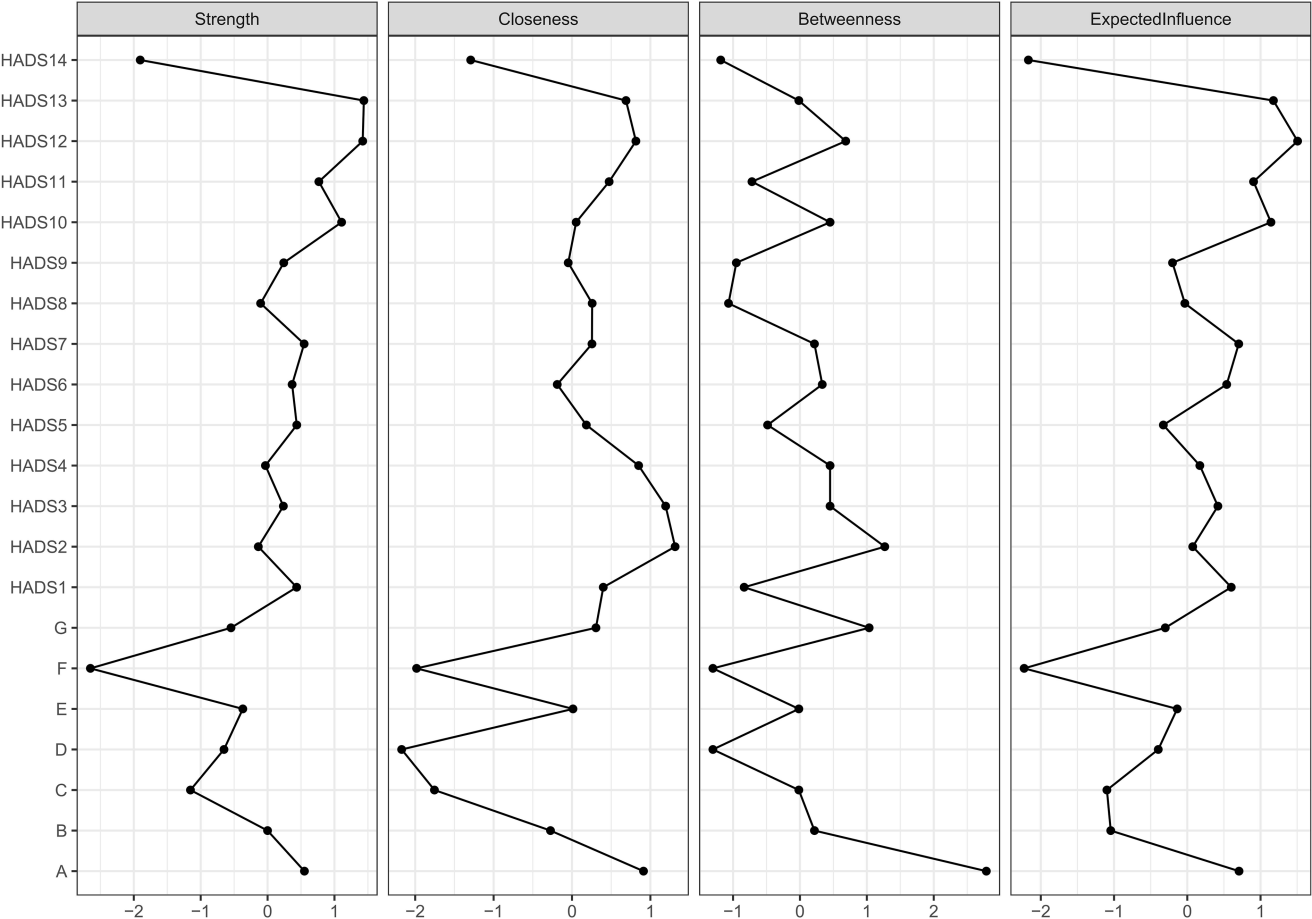
Measurement of centrality indices for the constellation of sleep quality-anxiety/depressive symptom among patients with CHF.

In the network centrality analysis (**Figure 2**), strength centrality, which quantifies a node’s total direct connections and reflects its overall activity and influence, identified the most central symptoms. The highest strength centrality was seen in HADS13 (sudden panic) and HADS12 (not looking forward with enjoyment to things) at 1.208 and 1.205, respectively, suggesting that these factors were most strongly correlated with other symptoms and that they represented pivotal nodes within the overall symptom network. Other high-strength nodes included HADS10 (“lost interest in appearance”, depression) and HADS11 (“restlessness”, anxiety), underscoring that both anxiety and depressive symptoms are highly interconnected within the network. Betweenness centrality, a metric that indicates how often a node falls on the shortest path between other nodes, highlighted symptoms with potential control over information flow. It was found that A (subjective sleep quality) and HADS2 (not enjoying the things that used to be enjoyed) exhibited the maximum betweenness centrality values, marking them as critical gatekeepers within the symptom network. The closeness centrality values for the majority of nodes (primarily the HADS symptoms) are clustered within a narrow range (approximately 0-1). Although the full scale of the metric is broader due to a few nodes with lower centrality, this clustering indicates that the core symptom network is uniformly connected, with most symptoms being roughly equidistant from one another, reflecting better global connectivity.

### Analysis of mediating symptoms in the sleep quality-anxiety/depressive symptom network

**Figure 3:**
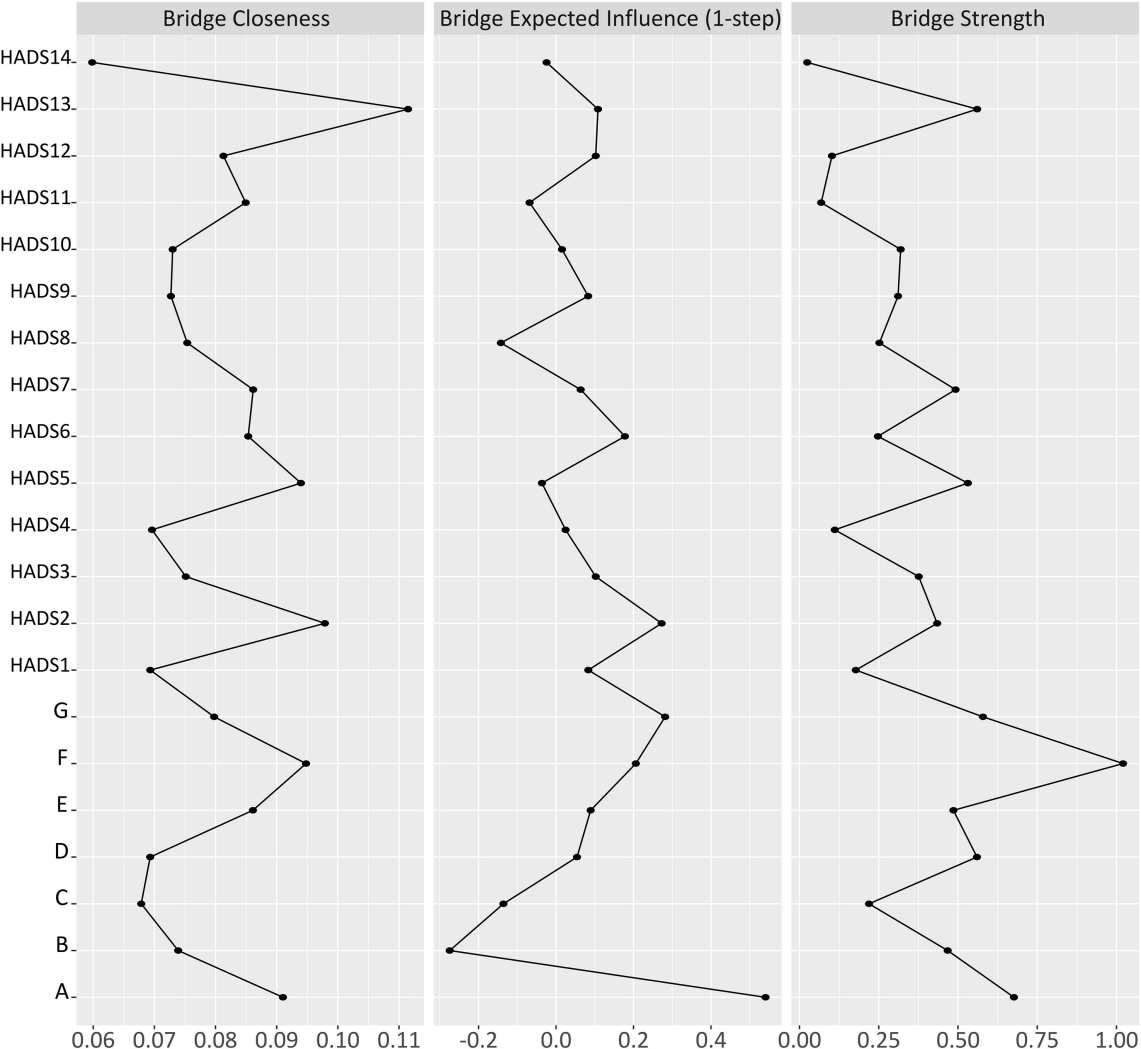
Bridge symptoms in the network of sleep quality-anxiety/depressive symptom in patients with CHF.

Bridge symptoms were defined as nodes exhibiting high bridge centrality, indicating their role in connecting different symptom communities. These symptoms were identified using the bridge expected influence (BEI) metric, which measures the sum of a node’s connection weights to other communities, thereby capturing its net potential influence. The analysis showed that Node A (subjective sleep quality) had the highest BEI value (0.541), and Node G (daytime dysfunction) had the second highest value (0.282) (**Figure 3**). These two symptoms were the most prominent connectors in the network, indicating strong associative pathways between sleep disorder symptoms and anxiety/depressive symptoms (**Figure 3**).

### Accuracy and stability of networks

**Figure 4:**
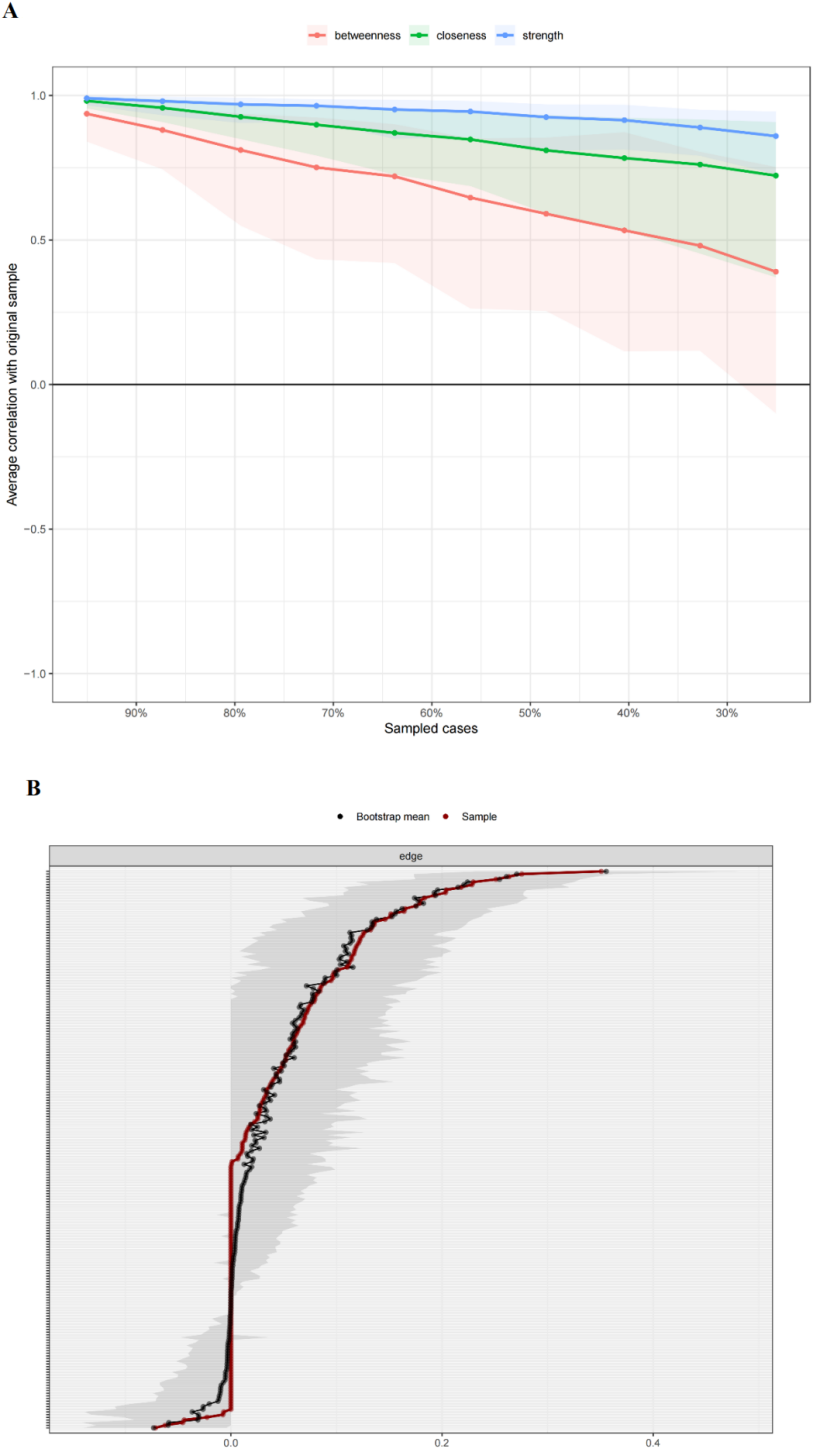
**(A)** Assessment of the stability of the sleep disorders-anxiety/depressive symptom network in patients with CHF. **(B)** Assessment of the accuracy of the edge weights in the sleep disordersanxiety/depressive symptom network (black lines represent the average edge weights calculated using a bootstrapping method, while red lines indicate edge weights associated with the sample used in this study).

The stability of the sleep quality-anxiety/depressive symptom network of patients with CHF was then evaluated. Assessment of the stability of the centrality indices (**Figure 4A**) showed a correlation coefficient of 0.749, indicating that the centrality indices were stable. A bootstrap method was used to analyze the accuracy of the weights of the network edges. The results shown in **Figure 4B** indicate that the 95% confidence interval (CI) for the edge weights exhibited a tight range, suggesting that the weight assessments were precise.

## Discussion

This study found a high co-occurrence of poor sleep quality (57.39%), anxiety symptoms (60.34%), and depressive symptoms (73.15%) in patients with CHF. These prevalence rates are substantially higher than the well-documented range in the literature, where anxiety affects approximately 16% to 52.4% and depression28.0% to 53.4% of CHF patients (28, 42–44). The disparity likely stems from the unique profile of our study population, which was characterized by a high prevalence of severe heart failure (total 87.7% in NYHA class III and class IV) and a prominent proportion of low-income individuals (69.0%). While the reliance on self-reported measures may introduce bias (e.g., PSQI and HADS) (45), and symptom overlap can complicate assessment, the primary contribution of this study lies in moving beyond prevalence. Through network analysis, we elucidate the specific interrelationships among these symptoms. These high levels of sleep impairment, anxiety, and depressive symptoms are strongly associated with poorer well-being in patients with CHF and, in line with existing literature (10, 46, 47), may be linked to adverse disease outcomes, suggesting the potential for a reinforcing interplay. Consequently, it is imperative for clinicians to look beyond cardiac function and prioritize the assessment and management of psychological health and sleep quality. Implementing comprehensive interventions that address this complex symptom burden is essential for improving prognosis, enhancing overall health outcomes (48, 49), and restoring patients’ capacity for self-management—a fundamental step toward improving their quality of life (50).

This study investigated the interrelationships among sleep quality and anxiety/depressive symptoms in Chinese patients with CHF using a network perspective, thus offering a new theoretical structure for understanding the complexity of mental well-being in this patient population. Research has shown that sleep, guilt, restlessness, irritability, and feeling afraid were the primary core symptoms in sleep quality-anxiety/depressive symptoms network of Chinese college students (51). The present study highlights that HADS13 (sudden panic) and HADS12 (not looking forward with enjoyment to things) emerged as central nodes within the overall symptom network. Emerging findings in psychocardiology show symptom clusters in CHF, with Bian et al. (52) identifying fatigue, sleep disturbance, and emotional distress, paralleling our findings of lack of enjoyment and panic. Zhao et al. (53) noted anxiety and depressive symptoms, highlighting “loss of interest” and “fear/panic” as key nodes, emphasizing threat- and loss-related feelings in heart failure. He et al. (54) found anhedonia, agitation, and somatic anxiety central in coronary heart disease, indicating psychological distress often revolves around mood depletion and anticipatory fear across cardiac populations. The identification of ‘not looking forward with enjoyment to things’ and ‘sudden panic’ as the most central symptoms is clinically insightful. In CHF, ‘not looking forward with enjoyment to things’ likely transcends a general loss of interest; it may be directly fueled by the grief of functional loss, social role contraction, and the perceived burden of a chronic illness, which can erode future-oriented pleasure and motivation. Conversely, ‘sudden panic feelings’ could represent the somatic manifestation of a very specific and pervasive fear in CHF—the fear of acute dyspnea, sudden deterioration, or imminent mortality. Their centrality suggests that in CHF, the emotional burden may be organized around themes of loss and threat. These symptoms could represent putative leverage points for psychological intervention, a possibility that requires confirmation in prospective studies. Having identified these central emotional drivers, elucidating the bridge symptoms that connect the sleep and psychological symptom clusters is equally crucial to understanding the integrated network.

The bridge function of ‘subjective sleep quality’ and ‘daytime dysfunction’ helps explain the observed correlations between the broader illness burden and psychological distress in CHF. ‘subjective sleep quality’ can be understood as a perceptual link: long-term physical discomfort and symptoms (e.g., nocturnal dyspnea) are strongly correlated with sleep disturbances, which are subjectively reported as poor sleep quality. This perception of poor sleep shows a notable correlation with heightened anxieties about health and decline. Similarly, ‘daytime dysfunction’ serves as a phenomenological link: the pervasive fatigue intrinsic to advanced CHF and its socioeconomic burden overlaps phenomenologically with the anergia core to depression and is also a commonly co-occurring feature of poor sleep.

The unpredictable progression of CHF is linked to heightened patient concerns about organ damage and mortality (55), a specific fear that corresponds to the core symptom ‘sudden panic feelings’ and is intertwined with the overall psychological stress that accompanies sleep disturbances. This creates a dense web of interconnections. Literature suggests that poor sleep is correlated with imbalances in neurotransmitters and compromised emotional regulation, which are linked to higher levels of anxiety and depressive symptoms (56). Correspondingly, elevated psychological stress shows a strong association with sleep disturbances. These observed interconnections point to a potential reinforcing interplay among sleep disturbances, emotional symptoms, and core physical symptoms.

Available reports show the pivotal function of bridge symptoms in both the maintenance and progression of comorbid mental disorders. These symptoms thus represent a critical focal point for the prevention and management of these diseases (57).The bridging function of sleep-related symptoms has also been underscored in recent cardiac network research. For instance, Bian et al. (52) reported that impaired sleep quality served as a pathway linking somatic discomfort and emotional distress clusters during the vulnerable phase of CHF, implying that bridge nodes may facilitate shifts between physical and psychological burden. Similarly, Zhao et al. (53) identified insomnia and concentration difficulties as central bridging elements connecting anxiety and depressive symptoms in CHF patients. In this study, subjective sleep quality and daytime dysfunction were identified as key bridge symptom which may serve as hubs to integrate core symptoms such as ‘use of sleeping medication’, ‘sudden panic’, and ‘not looking forward with enjoyment to things’. This cluster of interconnected symptoms is closely clustered with poorer mental health and sleep outcomes in patients with CHF. Notably, the bridge symptoms identified here differ from those reported in other conditions, such as ‘trouble relaxing’ and ‘feeling down’ as bridging symptoms between anxiety and depressive in patients with rheumatoid arthritis (58), suggesting the existence of disease-specific symptom pathways. Impaired cardiac function characteristic of CHF is accompanied by blood circulation. Insufficient blood supply to the brain may be linked to neurotransmitter imbalances and consequent alterations in brain function, which could serve as potential correlates of poor sleep quality and fear of injury (59). Thus, effective management in CHF should extend beyond optimizing cardiac function to include targeted attention to bridging symptom clusters identified in the present network analysis. Improving subjective sleep quality, in particular, may represent a practical and potentially impactful entry point for modifying the broader symptom network. Multimodal strategies—such as pharmacotherapy, cognitive-behavioral therapy, stress reduction, and mindfulness-based practices—could be employed to address this target. While these approaches hold promise for alleviating sleep deficits and mitigating associated psychological burdens, their utility as intervention priorities should be regarded as a hypothesis arising from current cross-sectional evidence and thus warrants confirmation in prospective and experimental studies (60, 61), before firm causal inferences or clinical recommendations can be established.

Building on this integrated framework, we propose that specific interventions could be tailored to the central symptoms identified in our network, as hypothetical priorities that warrant prospective testing before clinical application. Cognitive behavioral therapy (CBT) serves as a foundational intervention for both core symptom clusters, effectively addressing the irrational beliefs underlying anxiety (e.g., frightened feelings) and the negative thought patterns characterizing depression (e.g., feeling down) (62). Specifically, for the core symptom frightened feelings (‘sudden panic’), healthcare providers should offer clear explanations of CHF pathophysiology and treatment, which may enhance the patient’s sense of control and help mitigate health-related anxieties (63), alongside implementing targeted CBT strategies (64). Concurrently, for the core depressive symptom of ‘not looking forward with enjoyment to things’, CBT can be usefully supplemented with moderate exercise (65) as well as music (66) and art therapy, among other rehabilitative activities, to help shift focus away from negative feelings. At the same time, communication with family members should be strengthened, emphasizing the importance of cultivating a positive family atmosphere while providing care and companionship (67).

The bridge symptom of subjective sleep quality may serve as a potential intervention target within this symptom network. This hypothesis addresses the interconnectedness between psychological distress and sleep disturbance, and should be evaluated in future longitudinal and interventional studies before firm conclusions are drawn. To effectively address poor subjective sleep quality, a stepped-care approach is recommended. First, healthcare providers should deliver structured sleep education to cultivate healthy sleep habits. For patients experiencing significantly sleep impairment, pharmacological interventions may be considered following a thorough medical evaluation. Furthermore, the integration of relaxation training into the treatment plan can promote better sleep quality, while cognitive behavioral therapy may help modify maladaptive thoughts related to sleep (68).

### Strengths and limitations of the study

This study has several strengths, primarily its novel use of network analysis to explore the interrelationships between sleep quality, anxiety, and depressive symptoms in CHF patients, identifying central and bridge symptoms for targeted intervention. Several limitations should be considered. First, generalizability is limited by the single-center design and a sample over-representing severe cases (NYHA class III–IV) and individuals with lower socioeconomic status. Furthermore, the cross-sectional design precludes causal inference, and the use of self-reported measures may introduce bias. Finally, due to sample size constraints, this study estimated a single combined network. This approach prevents examination of how symptom interactions might differ across NYHA classes and, consequently, further limits the generalizability of our findings to the broader CHF population with varying disease severity. Therefore, the clinical implications of this study should be considered preliminary. Although interventions targeting the identified core and bridge symptoms could potentially alleviate the symptom network, their efficacy requires validation through future multi-center longitudinal studies incorporating objective measures.

## Conclusions

In conclusion, this study identified the key characteristics of the sleep quality-anxiety/depressive symptoms network in patients with CHF. Beyond identifying HADS12 (not looking forward with enjoyment to things) and HADS13 (sudden panic) as core symptoms within the network, it also revealed PSQI-A (subjective sleep quality) as the most prominent bridge symptom, connecting the sleep quality symptom cluster with the anxiety/depressive symptom cluster. The application of interventional measures simultaneously targeting these core and bridge symptoms may help disrupt the connections within the symptom network, break the observed vicious cycle, and reduce the overall symptom burden in patients. However, these potential intervention priorities should be regarded as preliminary hypotheses, and their effectiveness requires prospective confirmation in multi-center longitudinal and interventional studies to facilitate the precise treatment of related symptoms in patients with CHF. 

## Data Availability

The raw data supporting the conclusions of this article will be made available by the authors, without undue reservation.
